# Prone Positioning as a Potential Risk Factor for Deep Vein Thrombosis in COVID-19 Patients: A Hypothesis Generating Observation

**DOI:** 10.3390/jcm11010103

**Published:** 2021-12-25

**Authors:** Caroline E. Gebhard, Núria Zellweger, Catherine Gebhard, Alexa Hollinger, Leon Chrobok, David Stähli, Christof M. Schönenberger, Atanas Todorov, Markus Aschwanden, Martin Siegemund

**Affiliations:** 1 Intensive Care Unit, Department of Acute Medicine, University Hospital Basel, 4031 Basel, Switzerland; nuria.zellweger@usb.ch (N.Z.); alexa.hollinger@usb.ch (A.H.); leon.chrobok@stud.unibas.ch (L.C.); david.staehli@stud.unibas.ch (D.S.); c.schoenenberger@stud.unibas.ch (C.M.S.); martin.siegemund@usb.ch (M.S.); 2 Department of Clinical Research, University of Basel, 4056 Basel, Switzerland; 3 Department of Nuclear Medicine, University Hospital Zurich, 8091 Zürich, Switzerland; Catherine.Gebhard@usz.ch (C.G.); Atanas.Todorov@usz.ch (A.T.); 4 Center for Molecular Cardiology, University of Zurich, 8952 Schlieren, Switzerland; 5 Department of Angiology, University Hospital Basel, 4031 Basel, Switzerland; Markus.Aschwanden@usb.ch

**Keywords:** coronavirus, prone position, deep vein thrombosis, ARDS, obesity, blood viscosity, Virchow’s triad

## Abstract

Aims of the study: Virchow’s triad with stasis, activated coagulation, and endothelial damage is common in SARS-CoV2. Therefore, we sought to retrospectively assess whether the duration of prone position may serve as a risk factor for deep vein thrombosis in critically ill patients. Methods: In this single center retrospective study of a tertiary referral hospital, patients with acute respiratory distress syndrome (ARDS) due to COVID-19 pneumonia admitted to critical care underwent venous ultrasound screening for deep vein thrombosis (DVT). Data on DVT diagnosis, duration of prone positioning, demographic, respiratory, and laboratory parameters were retrospectively collected and compared between DVT and non-DVT patients. Results: 21 patients with ARDS from COVID-19 pneumonia were analyzed. DVT was detected in 11 (52%) patients (76.2% male, median age 64 (58; 68.5) years, median body mass index 31 (27; 33.8) kg/m^2^). In patients diagnosed with DVT, median prone ventilation had been maintained twice as long as compared to patients without DVT (57 (19; 72) versus 28 (0; 56.3) h, *p* = 0.227) on ICU day 5 with a trend towards longer prone position time (71 (19; 104) versus 28 (0; 73) h, *p* = 0.06) on ICU day 7. Conclusions: Prone ventilation and constitutional factors may constitute an additional risk factor for DVT in COVID-19 patients. Since recent studies have shown that therapeutic anticoagulation does not impact the occurrence of thromboembolic events, it may be worthwhile to consider mechanical factors potentially affecting blood flow stasis in this high-risk population. However, due to the limited number of patients, our observations should only be considered as hypothesis-generating. Future studies, sufficiently powered and preferably prospective, will be needed to confirm our hypothesis.

## 1. Introduction

Coronavirus disease-2019 (COVID-19) is associated with a high burden of venous thromboembolic events (VTE) in critically ill patients [1]. Deep vein thrombosis (DVT) is the most common manifestation of coagulopathy and diagnosed in up to 84% of severely ill patients infected with severe acute respiratory syndrome coronavirus-2 (SARS-CoV2) despite prophylactic anticoagulation [2]. The etiology of VTE in COVID-19 patients is considered multifactorial, determined by excessive systemic inflammation, vascular endothelial dysfunction, and complement activation leading to a pro-coagulant state. In addition, hypoxia, constitutional factors, hyperviscosity, and blood-flow and stasis may further promote the occurrence of VTE [3]. In COVID-19 acute respiratory distress syndrome (ARDS), prone position (PP) was frequently applied. During prone ventilation, the patient’s position remains nearly unchanged with minimal movements limited to the head and limbs. While several studies have addressed the higher risk of DVT in critically ill patients, PP as a potential contributor to blood-flow changes has not been considered as a risk factor in these trials [4,5,6]. Following the urgent call for close ultrasound screening for the early detection of DVT [7], we aimed to evaluate whether the application and duration of prone position may serve as an additional risk factor for DVT in critically ill patients with SARS-CoV2 admitted to the intensive care unit (ICU) and to raise a new aspect of DVT genesis in ARDS patients.

## 2. Materials and Methods

### 2.1. Patient Selection

We conducted a retrospective chart review of all patients admitted to our ICU with severe SARS-CoV2 between 03 April 2020 (start date of routine ultrasound screening) and 21/07/2020 (last COVID-19 patient admitted during the first wave). All data were collected from the electronic patient files. Patients aged < 18 years, not mechanical ventilated or with an ICU stay < 48 h were excluded from the analysis.

The present study was approved by the local ethic committee of Northwest and Central Switzerland (#2020−01355). 

### 2.2. Baseline Demographics, Disease Severity and Outcome

Age, BMI, sex, start date of invasive ventilation, and ICU death were collected from the electronic patient file. Coagulation and inflammatory parameters, the Simplified Acute Physiology Score (SAPS) II were collected within the first 24 h after ICU admission. Active cancer diseases or neoplasia under treatment were gathered from the past medical history. 

### 2.3. Ultrasound DVT Screening, DVT Diagnosis and Thromboembolic Events

All patients had undergone a routine complete duplex ultrasound examination for early detection of DVT by a senior angiologist. Lower extremities and cervical venous vessels had been examined with repetitive visits every other day including central line insertion sites. Diagnosis of vein thrombosis was based on an intraluminal hyperechoic filling of the veins in B-mode [8]. Data were retrospectively collected from the electronic reports, including normal and pathological findings of the vein segments of the lower and upper extremities. DVT ultrasound screening was stopped when DVT was diagnosed and/or a therapeutic dose of anticoagulation was newly implemented. In addition, data on all thromboembolic events, including pulmonary embolism, ischemic stroke, mesenteric ischemia, peripheral arterial occlusion, or myocardial infarction confirmed by imaging or diagnostic interventions, were collected. 

### 2.4. Initiation/Duration of Prone Positioning, Respiratory Parameters and Fluid Balance

Early PP was applied according to current evidence in patients with severe ARDS in COVID-19 patients [9]. Prone positioning was initiated for respiratory failure with a P/F ratio < 150 mmHg or if a P/F ratio of higher than 150 mmHg was only achievable with a plateau pressure above 30 mbar or a driving pressure > 15 mbar. Duration was usually two shifts meaning 16 h. Prone positioning was stopped when the FiO_2_ support remained stable on two consecutive days after return to supine position. Data on total PP time, average and maximum positive end-expiratory pressure (PEEP) were collected up to ICU day 5 and 7. These timepoints were chosen as the average day (median: day 5; mean: day 7) of DVT diagnosis and used to compare time in prone position in patients with and without DVT.

P/F ratios were calculated from lowest PaO_2_ with the corresponding FiO_2_ and the highest FiO_2_ with the corresponding PaO_2_ within the first 24 h after ICU admission, on day 5 and day 7. The worst P/F ratio was chosen for each timepoint. 

Cumulative fluid balance on day 5 and 7 was calculated from the cumulative intake, such as enteral nutrition, intravenous fluids, and transfusions, while fluid losses included urine output, GI losses and bleeding. 

### 2.5. Anticoagulation Treatment and Bleeding Complications

Anticoagulation treatment was applied with either unfractionated or low-molecular weight heparin and the average dose collected up to ICU day 5. Prophylactic anticoagulation was conducted with dalteparin 5000 or 7500 IU/24 h SC or UFH heparin 10,000–15,000 IU/24 h according to body weight. High-prophylactic anticoagulation was defined as dalteparin 100–150 IU/kg SC or UFH heparin with an anti-Xa target of 0.15–0.25 U/ml. Therapeutic anticoagulation was defined as dalteparin 200 IU/kg or UFH heparin with an anti-Xa target of 0.250.65 U/mL. Therapeutic anticoagulation was only applied in the case of pre-existing long-term anticoagulant therapy or if newly indicated (new-onset atrial fibrillation, thromboembolic events). Bleeding complications were either defined as major or non-major bleeding. According to the International Society on Thrombosis and Haemostasis, overt bleeding was defined as follows: hemoglobin fall > 20 g/L, fatal bleeding, symptomatic bleeding in critical areas or organs, need for blood transfusion of more than two units of packed red cells. All non-major bleeding not qualifying for one of the above categories were considered as minor bleeding. 

### 2.6. Statistics

Descriptive statistics included percentages for categorical variables and median (interquartile range) for continuous variables. A Kolmogorov–Smirnov test was used to determine the normality of data distribution. Variables were compared between groups using the Mann–Whitney test or Chi^2^/Fisher’s exact test, as appropriate. The median and mean time to DVT development was calculated and set as time-points for data collection (e.g., PP time) in patients without events. A two-sided *p*-value < 0.05 was regarded as statistically significant. (IBM^®^ SPSS^®^ statistical package, version 26). 

## 3. Results

### 3.1. Baseline Demographics, Disease Severity and Outcome

21 patients (median age 64 (58; 68.5) years, 76.2% male, body mass index (BMI) 31 (27; 33.8) kg/m^2^) were included into the analysis. Median age (DVT 62 (58; 68) years versus non-DVT 64 (58; 69) years, *p* = 0.654) and BMI (DVT 32 (31; 34) kg/m^2^ versus non-DVT 30 (26.5; 33.7) kg/m^2^, *p* = 0.387) did not differ between groups. No differences in disease severity estimated by the SAPS II (DVT 54 (44; 66); non-DVT 56.5 (41; 86), *p* = 0.756) was observed. Median P/F ratio upon ICU admission was 100 (69; 113) mmHg in patients without DVT and 95 (75; 121) mmHg in patient with diagnosis of DVT, *p* = 0.901. No differences in total ICU stay and ICU death were observed. Active cancer diseases or neoplasia were present in two patients with DVT (one with oropharyngeal cancer, one with follicular lymphoma) and in one patient without DVT (follicular lymphoma), *p* = 1.000. 

### 3.2. DVT Diagnosis, Anticoagulation Regimen and Adverse Events

DVT was detected in 11 patients, while 10 patients remained free of DVT. All patients were sedated and mechanically ventilated at the time of assessment. Four patients in the DVT group showed central vein catheter (CVC) associated thrombosis (36.4%), three of them in the internal jugular vein and one in the common femoral vein. In three out of four patients, a concomitant lower extremity DVT was present. All patients received thromboprophylaxis upon ICU admission. More patients in the DVT group were under high-prophylactic or therapeutic anticoagulation (*n* = 6, 54.5%) compared to patients in the non-DVT group (*n* = 4, 40%). However, no significant difference in anticoagulation regimen was observed (*p* = 0.752). In two patients of the DVT group, PE occurred. In one patient, subsegmental PE was confirmed the same day of DVT diagnosis. The second patient who was under a prophylactic dose of LMWH suffered from central PE on ICU day three and DVT was subsequently confirmed. The patient died from multiorgan failure. In each group, two patients died during ICU course. 

### 3.3. Prone Position Time, Respiratory Parameters and Fluid Balance

In median PP time, a trend towards a higher prone time in patients with DVT compared to non-DVT patients upon ICU day 7 (71 (19; 104) h versus 28 (0; 73) h, *p* = 0.006) was observed, while we found no differences in PP time summarized up to day 5 in DVT patients compared to non-DVT patients (57 (19; 72) h versus 28 (0; 56.3) h, *p* = 0.227). Median average and maximum PEEP up to day 5 and 7 and the P/F ratio on day 5 and 7 did not differ between groups. Although there was no significant difference between the cumulative fluid balance on day 5 and 7, the median cumulative fluid balance in DVT patients on day 7 (475 (−2101; 3781) was less than a third compared to non-DVT patients 1567 (−42.5; 5446), *p* = 0.605. Table 1 summarizes demographics, clinical, laboratory, and outcome parameters.

## 4. Discussion

In this small retrospective analysis in patients with confirmed COVID-19, we found that DVT occurred in 52% of our patients. In patients diagnosed with DVT, a trend towards longer PP time for acute respiratory distress syndrome on day 7 has been observed compared to patients without DVT.

Multiple studies have been conducted to assess the high prevalence of thromboembolic events in COVID-19 diseases despite standard pharmacologic thromboprophylaxis. [1,10]. Most recent results from a large randomized, multicenter trial could not show any advantage of therapeutic anticoagulation in terms of thromboembolic events and outcome [11]. As already taught by Rudolf Virchow in 1848, a triad of factors consisting of blood flow stasis, activated coagulation, and endothelial damage contributes to the genesis of thrombosis [12,13]. Excessive systemic inflammation and prothrombotic phenotypes are common features in SARS-CoV2 and associated with a higher risk of adverse outcomes in COVID-19 patients. [14,15,16]. While we did not observe significant differences in patients with DVT and without DVT development in coagulation and inflammatory parameters, a doubling of PP time was found in patients with DVT. Prone positioning is typically performed in deeply sedated patients with additional use of neuromuscular blockade in case of relevant patient-ventilator asynchrony. Comparable settings can be observed in the operating room in patients undergoing spinal surgery in PP. In these patients, the incidence of thrombotic complications after 2–4 h was described to occur in up to 12% of patients [17]. Considering the fact that PP is usually performed over 16 hours in patients with ARDS according to current evidence [18], a potential risk for the development of DVT can be estimated. In PP, abdominal compression leads to an increase in intraabdominal pressure with consecutive compression of the iliac and femoral veins, which, in turn, may decrease blood flow velocity even leading to blood stasis [19]. Obesity may aggravate this phenomenon and is frequently observed in critically ill COVID-19 patients. 

Positive pressure mechanical ventilation with increased levels of PEEP as frequently applied in ARDS patients may further impair venous backflow particular from upper extremity veins [5]. A previous study investigated the impact of positive end-expiratory pressure on the development of upper extremity DVT without finding an association in supine positioned patients [20]. However, prone ventilation with face-down position was linked to mechanical compression and possible kinking of the internal jugular vein [21], which might contribute to DVT development in prone positioned patients. Importantly, four out of 11 patients showed catheter related thrombotic events, three of them in the jugular vein. Furthermore, restrictive fluid management, as often applied in ARDS patients as well as inflammatory acute phase reactants, may additionally trigger VTE occurrence by altering blood viscosity [22]. In our patients, median cumulative fluid balance was lower in the DVT group on day 7, however, not significantly different between groups. 

There are several limitations to this study: First, this is a single-center and retrospective analysis with a pure observational design and thus carries a limited generalizability of its results and a potential for referral bias. Second, the study lacks statistical power to detect an interaction between DVT development and time of PP (estimated required sample size = 220) and should therefore only be considered hypothesis generating. Due to the small sample size, effect estimation based on multivariate regression would not be stable and thus was not performed. Third, a control group consisting of non-COVID-19 ARDS patients was not included in our analysis. Thus, it remains unclear whether COVID-19 and non-COVID ARDS patients being prone positioned have an equal risk to develop DVT. Fourth, due to the limited number of patients with routine DVT screening and the high rate of prone position in COVID-19 patients, we were not able to provide a sufficient number of patients with DVT screening not undergoing prone position. Therefore, we lack statistical power to compare the correlation of DVT between groups of patients with and without prone position. However, in our study cohort, five patients did not undergo prone position. In two of them, DVT was detected during ultrasound screening. Fifth, based on the limited evidence at the beginning of the pandemic, anticoagulation strategies were not uniformly applied in the included patients. Nevertheless, this did not result in significant differences between DVT and non-DVT patients. 

## 5. Conclusions

Although we were not able to clearly demonstrate a significant difference in prone position time between groups prior to DVT diagnosis, prone position may contribute as an additional risk factor to thrombogenesis by increasing blood flow stasis. Given the small patient population studied and insufficient power to reach statistical significance, our data should only be considered hypothesis-generating and serve as a catalyst for future, sufficiently powered studies. Meanwhile, it may benefit the physician to consider mechanical and constitutional determinants as possible contributors for DVT development in COVID-19 patients.

## Figures and Tables

**Table 1:** Baseline demographics, clinical, laboratory and outcome parameters in patients with coronavirus disease 19 undergoing ultrasound screening for DVT.

	Baseline Demographics, Disease Severity and Outcome Parameters							
	All (*n* = 21)		Patients without DVT (*n* = 10)		Patients with DVT (*n* = 11)		*p*-Value	
Age (years) median (IQR)	64 (58; 68.5)		64 (58; 69)		62 (58; 68)		0.654	
Male *n* (%)	16 (76.2%)		8 (80)		8 (72.7)		1.000	
BMI (kg/m^2^) median (IQR)	31 (27; 33.8)		30 (26.5; 33.7)		32 (31; 34)		0.387	
SAPS II score median (IQR)	54 (37; 69)		56.5 (41; 86)		54 (44; 66)		0.756	
Active cancer *n* (%)	3 (14.3)		1 (9.1)		2 (20)		1.000	
Start mechanical ventilation ICU day	1 (1; 1)		1 (1; 1.25)		1 (1; 1)		0.586	
P/F ratio ICU day 1 (mmHg) median (IQR)	100 (74; 114)		102 (69; 113)		95 (75; 121)		0.901	
Total ICU-LOS (days) median (IQR)	16 (10.5; 23)		12.5 (9.5; 16.8)		19 (12; 31)		0.118	
ICU death *n* (%)	4 (19)		2 (20)		2 (18.2)		1.000	
	**Adverse Events and Anticoagulation**							
Pulmonary embolism *n* (%)	2 (9.5)		0 (0)		2 (18.2)		0.476	
Upper extremity DVT *n* (%)	3 (14.3)		0 (0)		3 (27.3)		0.214	
Bleeding complication *n* (%)	3 (14.3)		1 (10) (major bleeding)		2 (18.2) (minor bleedings)		1.000	
Prophylactic anticoagulation before day 5/7 *n* (%)	11 (52.4)		6 (60)		5 (45.5)		0.725	
High-prophylactic anticoagulation before day 5/7 *n* (%)	4 (19)		1 (10)		3 (27.3)			
Therapeutic anticoagulation before day 5/7 *n* (%)	6 (28.6)		3 (30)		3 (27.3)			
	**Inflammatory and Coagulation Parameters upon ICU Admission**							
Platelets (×10^9^/L) median (IQR)	201 (174; 297)		190 (152; 212)		214 (188; 346)		0.051	
INR median (IQR)	1.1 (1.1; 1.3)		1.1 (1.0; 1.3)		1.1 (1.1; 1.3)		0.705	
Fibrinogen (g/L) median (IQR)	5.3 (3.6; 6.3)		4.2 (3.4; 5.6)		6.2 (4.3; 6.7)		0.114	
aPTT (s) median (IQR)	33 (29; 36)		35 (31; 36)		31 (29; 35)		0.197	
D-Dimers (μg/mL) median (IQR)	1.3 (0.6; 4.3)		1.1 (0.6; 1.5)		1.9 (1.3; 5.4)		0.114	
CRP (mg/L) median (IQR)	146 (69; 215)		146 (48; 168)		149 (96; 244)		0.314	
Ferritin (µg/L) median (IQR)	1501 (997; 2156)		1764 (1146; 4378)		1194 (898; 2176)		0.282	
Leucocytes (10^9^/L) median (IQR)	7.1 (5.2; 10.1)		5.4 (2.9; 8.9)		8.9 (5.6; 10.8)		0.085	
Lymphocytes (10^9^/L) median (IQR)	0.58 (0.38; 0.78)		0.65 (0.3; 0.74)		0.49 (0.38; 1.41)		0.973	
	**Prone Position Time and Respiratory Parameters on ICU Day 5 and 7**							
	**ICU day 5**				**ICU day 7**			
	**All (*n* = 21)**	**Patients without DVT (*n* = 10)**	**Patients with DVT (*n* = 11)**	***p*-Value**	**All (*n* = 21)**	**Patients without DVT (*n* = 10)**	**Patients with DVT (*n* = 11)**	***p*-Value**
Patient in prone position *n* (%)	16 (76.2)	7 (70)	9 (81.8)	0.635	16 (76.2)	7 (70)	9 (81.8)	0.635
Patient without prone position *n* (%)	5 (23.8)	3 (30)	2 (18.2)		5 (23.8)	3 (30)	2 (18.2)	0.635
Prone position time (hours) median (IQR)	43 (8; 68)	28 (0; 56.3)	57 (19; 72)	0.227	43 (9; 80)	28 (0; 73)	71 (19; 104)	0.060
P/F ratio ICU (mmHg) median (IQR)	146 (98; 163)	155 (112; 170)	120 (95; 151)	0.274	135 (119; 171)	147 (126; 182)	127 (113; 160)	0.323
Average PEEP until day 5/7 (mbar) median (IQR)	12.7 (12; 14.6)	12.9 (11.8; 14.3)	12.7 (12.1; 15)	0.654	12.8 (11.4; 14.3)	13 (11.1; 14.1)	12.4 (11.6; 14.7)	0.756
Maximum PEEP until day 5/7 (mbar) median (IQR)	15 (14; 15.5)	15 (13.8; 15)	15 (15; 18)	0.1	15 (14.5; 16.5)	15 (14; 15.3)	15 (15; 18)	0.319
Cumulative fluid balance (mL) median (IQR)	1882 (−98; 4820)	1678 (349; 6490)	2112 (−1651; 1315)	0.468	1425 (−552; 4505)		475 (−2101; 3781)	0.605

All results are given in median (IQR) or number (percentages). aPTT, activated partial thromboplastin time; BMI, body mass index; CRP, C-reactive protein; DVT, deep vein thrombosis; ICU, intensive care unit; INR, international normalized ratio; LOS, length of stay; mmHg, millimeter of mercury; PEEP, positive end-expiratory pressure; P/F ratio, PaO_2_/FiO_2_ ratio; SAPS, simplified acute physiology score.

## Data Availability

The data presented in this study are available on request from the corresponding author. The data are not publicly available due to privacy and ethical reasons.
